# NK cell defects: implication in acute myeloid leukemia

**DOI:** 10.3389/fimmu.2023.1112059

**Published:** 2023-05-09

**Authors:** Selma Z. D’Silva, Meenakshi Singh, Andrea S. Pinto

**Affiliations:** ^1^ Transplant Immunology and Immunogenetics Lab, Advanced Centre for Treatment, Education and Research in Cancer (ACTREC), Tata Memorial Centre, Navi Mumbai, India; ^2^ Homi Bhabha National Institute, Mumbai, India

**Keywords:** natural killer cells, immunotherapy, AML, CAR-NK, BiKEs, TriKEs

## Abstract

Acute Myeloid Leukemia (AML) is a complex disease with rapid progression and poor/unsatisfactory outcomes. In the past few years, the focus has been on developing newer therapies for AML; however, relapse remains a significant problem. Natural Killer cells have strong anti-tumor potential against AML. This NK-mediated cytotoxicity is often restricted by cellular defects caused by disease-associated mechanisms, which can lead to disease progression. A stark feature of AML is the low/no expression of the cognate HLA ligands for the activating KIR receptors, due to which these tumor cells evade NK-mediated lysis. Recently, different Natural Killer cell therapies have been implicated in treating AML, such as the adoptive NK cell transfer, Chimeric antigen receptor-modified NK (CAR-NK) cell therapy, antibodies, cytokine, and drug treatment. However, the data available is scarce, and the outcomes vary between different transplant settings and different types of leukemia. Moreover, remission achieved by some of these therapies is only for a short time. In this mini-review, we will discuss the role of NK cell defects in AML progression, particularly the expression of different cell surface markers, the available NK cell therapies, and the results from various preclinical and clinical trials.

## 1 Introduction

### 1.1 Acute myeloid leukemia

Acute Myeloid Leukemia (AML) is a type of blood cancer that affects the cells of the myeloid lineage (1). It is the most common type of blood cancer with poor survival due to the aggressiveness of the disease and high relapse rates (2, 3). To date, limited therapeutic options are available to treat AML (1, 3). Chemotherapy is the primary treatment given to AML patients. Depending on the risk of the disease, some patients may undergo Hematopoietic Stem Cell Transplantation (HSCT) (2, 3).

In the past few years, novel treatment options have been explored to improve the survival of patients suffering from AML. Targeted gene therapies, cytokine therapy, immunotherapy, etc., are some of the upcoming novel therapeutic options. Of these, immunotherapy is a promising strategy that explores the use of the patient’s immune system to kill leukemic cells (2–4).

### 1.2 NK cells and its receptors

Natural Killer cells play an essential role in innate immunity, and their anti-leukemic properties have been explored in the past few years (5). This anti-leukemic activity is driven by receptors on their surface, which transduces either activating or inhibitory signals. Most of these receptors belong to Killer Immunoglobulin-like receptors (KIRs), natural cytotoxicity receptors (NCRs) or NKG2 (Natural Killer Group 2) receptors. A balance between the activating and inhibitory receptors drives NK cell cytotoxicity. KIRs, NCRs and NKG2 can either activate NK cells by signaling through their immune receptor tyrosine-based activating motifs (ITAMs) or inhibit NK cells by signaling through their immune receptor tyrosine-based inhibitory motifs (ITIMs). These receptors need to recognize their cognate ligands to achieve their cytotoxic potential.

The KIR genes are present on chromosome 19q13.4. 16 KIR genes encode for the KIR receptors, namely KIR 2DL1, 2DL2, 2DL3, 2DL4, 2DL5, 2DS1, 2DS2, 2DS3, 2DS4, 2DS5, 3DL1, 3DL2, 3DL3, 3DS1 and two pseudogenes 2DP1, 3DP1 (5, 6). Inhibitory KIRs have a long cytoplasmic tail and hence are denoted as ‘L,’ whereas activating receptors have a short cytoplasmic tail and are denoted as ‘S.’Two KIR haplotypes, A and B, are determined by an individual’s KIR gene content. The A haplotype is inhibitory since it has more inhibitory KIR genes (KIR3DL1, KIR3DL2, KIR3DL3, KIR2DL1, KIR2DL3, KIR2DL4) and one activating KIR gene (KIR2DS4). On the other hand, the B haplotype is said to be an activating haplotype since it has more activating KIR genes (KIR2DS1, KIR2DS2, KIR2DS3, KIR2DS5, KIR3DS1) in addition to the inhibitory KIR genes (KIR3DL2, KIR3DL3, KIR2DL1, KIR2DL2, KIR2DL4, KIR2DL5A, KIR2DL5B) (7). The B haplotype is associated with lower relapse rates as it carries more activating KIR genes (8). KIR gene can further be divided into centromeric (Cen) and telomeric (Tel) regions to calculate the KIR B content score which can range from 0-2 B-content motifs for each of the centromeric and telomeric regions (i.e. AA, AB, or BB) (9). The haplogroup, which does not carry any of the B-content motifs is designated as CenAA/Tel-AA, whereas haplogroups that have scores ranging from 1 to 4, are designated as combinations of the following haplotypes CenAB/CenBB/TelAB/TelBB. The ligands for KIRs are the HLA Class I molecules (Bw4, C1, and C2). The inhibitory KIR2DL1 and activating KIR2DS1 recognize the C2 group alleles (possessing Lysine at position 80), the inhibitory KIR2DL2/3 and activating KIR2DS2/3 identify the C1 group alleles (having Asparagine at position 80), KIR3DL1 recognizes the HLA B alleles (with exceptions to B*13:01 and B*13:02) in the Bw4 group and KIR3DL2 identifies the HLA A*03/A*11 alleles (10). In the event of an inhibitory KIR receptor-ligand match, the NK cell activity is inhibited, leading to self-tolerance, whereas, in the case of an activating KIR receptor-ligand match, the NK cell activity is enhanced, leading to an increase in graft versus leukemia effect in the event of a transplant. Downregulation of the HLA ligands is an immune escape mechanism often used by tumor cells (11).

Among the NCRs, the activating NCRs are NKp30, NKp44, and NKp46, the ligands for which include various viral hemagglutinins (12–14), human CMV pp65 protein (15), and ligands such as B7-H6 and BAG6 which are expressed on the tumor cell surface. The NKP46 gene is very close to the Killer immunoglobulin-like receptor family on the human chromosome 19 (19q13.42) (16, 17). NKp44 and NKp30, on the other hand, are located in the MHC Class III region of Chromosome 6 (18, 19). NCRs such as NKp30 and NKp46, which were initially thought to be expressed only on resting NK cells (19– 20), has more recently been reported to be present on some T cells (Vd1 þ T cells for NKp30 and αβ T cells and δγ T cells for NKp44) and NK like cells (21, 22) where they perform similar functions as in NK cells. NKp44, on the other hand, is expressed only in activated NK cells. NCRs bind to their corresponding ligands, increasing cytotoxic activity and cytokine release by NK cells (23).

There are 7 members in the NKG2 family namely NKG2A, NKG2B, NKG2C, NKG2D, NKG2E, NKG2F and NKG2H. These transmembrane glycoproteins are encoded by genes in the NK complex present on chromosome 12. Of these 7 members, NKG2A, NKG2B (mRNA splice variant of NKG2A), NKG2C, NKG2E and NKG2H (mRNA splice variant of NKG2E) form heterodimers with CD94. NKG2F is expressed intracellularly (24).

**Figure 1:**
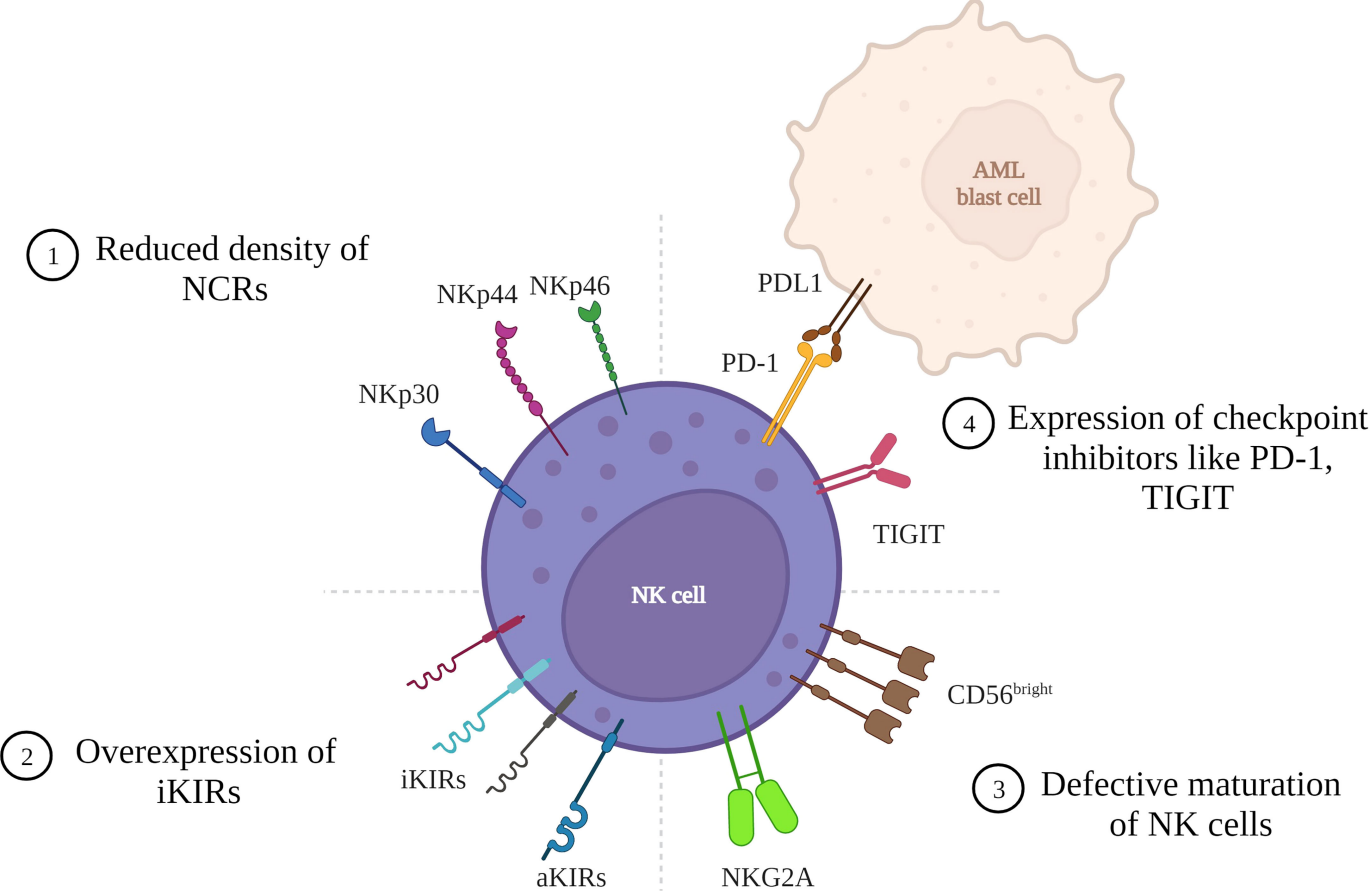
Mechanisms of tumor cell escape in AML due to NK cell defects. AML cells can evade tumor surveillance due to various defects in the NK cell that lead to reduced NK cell cytotoxicity: 1) Reduced density of natural cytotoxicity receptors (NCRs) on NK cell surface leading to a NCR^dull^ phenotype with lower cytolytic activity. 2) Overexpression of inhibitory Killer immunoglobulin like receptors resulting in higher inhibition of cytotoxicity. 3) Defective maturation of NK cells with majority cells with hypomature profile expressing CD56^bright/dim^ KIRs^-^ CD57^-^. 4) Expression of checkpoint inhibitors like PD-1 and TIGIT resulting in cells with reduced proliferative potential and lower cytotoxic and cytokine-producing capabilities. (Figure created in BioRender).

NKG2A is an inhibitory receptor (25) CD56^bright^ NK cell subsets show a higher expression of NKG2A than CD56^dim^ NK cells (26). CD94/NKG2A recognizes its ligand, HLA-E, a non-classical HLA molecule and transmits inhibitory signals *via* ITIMs present in the cytoplasmic tails thus inhibiting NK cell activity (24–26). NKG2A is overexpressed on the NK cells of AML patients which is associated with poor remission (26, 27). NKG2C is an activating receptor that is expressed on NK cells during the later stages of maturation (25). It binds to its ligand HLA-E with lower affinity than NKG2A. NKG2C interacts noncovalently to DAP12 containing ITAMs. NKG2D is an activating receptor expressed as a homodimer on NK cells. It binds to its ligand MICA/B and ULBP (also known as NKG2D-L) (28). NKG2D interacts with 2 dimers of DAP10 which recruits phosphatidylinositol-3 kinase (PI3 kinase) thus activating further signaling and NKG2D associated cytotoxic response. Tumor cells tend to shed NKG2D thus facilitating tumor escape (24, 25). NKG2D is underexpressed on NK cells of AML patients (27). NKG2D releases signals to activate the immune system by binding to the NKG2D-L present on AML cells and lyses the leukemic cells. However, in an event of downregulation of NKG2D-L tumor cells evade surveillance facilitating tumor progression (28). This NKG2D-NKG2DL axis is involved in clearing tumor cells in the early phases of cancer development. There are various mechanisms by which tumor cells escape NK-mediated cytotoxicity (**Figure 1**). These could be due to numerical defects in functional NK cells, defective expression of KIR and NCR receptors on NK cells, and defective maturation of NK cells, among others. This tumor evasion, if left unchecked, leads to the progression of the disease.

## 2 NK cell defects

### 2.1 Numerical defects

One factor that correlates with AML disease progression is the anti-leukemic activity exhibited by the NK cells, which also depends on the number of functionally active NK cells present. The number of NK cells is lowest during active disease, i.e., at the time of diagnosis and in the event of disease relapse. In contrast, it increases when the patient is in remission (29, 30). Reconstitution of higher NK cells post HSCT has been associated with better 2 year OS, lower rates of CMV reactivation (31), lower 2 year relapse risk (32), lower non relapse mortality, higher progression free survival (33). Conversely, low NK cell counts post 60 days of HSCT was associated with higher relapse risk (34).

### 2.2 NK receptors expression in AML vs. healthy controls

#### 2.2.1 Natural cytotoxicity receptors (NCR)

The activating NCRs are germline-encoded receptors that are immunoglobulin-like class I transmembrane molecules. They are essential in NK cell cytotoxicity against virus-infected and tumor cells. The brightness of immunofluorescence measures the density of NCRs. Most healthy donors express high densities of NCR exhibiting the NCR^bright^ phenotype, whereas those that carry the NCR^dull^ phenotype express a low density of NCRs (35, 36). There is a correlation between NCR expression and NK cell-mediated cytotoxicity and hence a correlation of NCR expression with leukemia response. NCR^bright^ NK cells display vigorous cytolytic activity compared to the NCR^dull^ NK cells that lack cytolytic activity (36). Various studies have shown that in most AML patients, the cell surface expression of NCRs is downregulated, thus exhibiting the NCR^dull^ phenotype, affecting the NK cell function and cytokine production. Another mechanism by which leukemic blast cells escape immune surveillance is lowering the expression of NCR ligands on their cell surface, thus preventing the engagement of NCRs with their respective ligands required to activate NK cell-mediated target lysis (35–37).

Studies in AML patients suggest better outcomes and more prolonged remission in patients with NK cells having higher cytolytic activity (38–41). Fauriat et al. (34) studied NCR recovery in AML patients who achieved complete remission (CR). They compared the NCR expression on NK cells of these patients at diagnosis and after treatment. They concluded that there was a partial recovery of NKp30 and NKp46 in these patients suggesting a direct correlation between NCRs and leukemic cells.

#### 2.2.2 KIR expression

In the past few years, researchers have sought to investigate the association of KIR and HLA genes with AML. These studies focus on assessing the KIR genes in AML patients and their association with post-transplant complications such as relapse, overall survival (OS), transplant related mortality (TRM) (5, 42). Daniele K.S. et al. (5) compared the KIR genes and haplotype frequency in patients suffering from various hematological malignancies with healthy individuals and observed that the frequency of the inhibitory genes - KIR2DL2 and KIR2DL5 and activating genes - KIR2DS1, KIR2DS2, and KIR2DS3 was more frequent in the controls than in the patients. Moreover, the frequency of KIR2DS3 was higher in AML patients than in ALL patients. The haplotype analysis suggested that haplotype A (more inhibitory) was more frequent in patients than in the controls. Victoria PV et al. (43) also reported a higher frequency of KIR2DL5A in the control group than in AML patients. Another study exploring the association of different AML risk groups with KIR and HLA genotypes observed that the Cen-AB/Tel-AB combination (B content =2), activating gene KIR2DS2 and the Bw4-80I and HLA-C2 allotype, was more frequent in AML patients than in the controls (42). Although these are a few individual studies, more extensive cohort studies are needed to assess the exact role of NK receptor expression profile in relation to AML disease progression.

### 2.3 Maturation of NK cells

There are two distinct Natural killer cell groups based on CD56 cell surface expression: 90% belong to the CD56^dim^ CD16^high^ group and are cytotoxic, and 10% belong to the CD56^bright^ CD16^dim/neg^ group and exhibit a more immunomodulatory role by releasing different cytokines (44). The maturation process of NK cells starts from CD34^+^ hematopoietic progenitor cells in the bone marrow, wherein cytokines like IL-15 and c-kit ligand, and flt-3 ligand help them reach their full potential from an NK cell progenitor (CD34^dim^/CD117^-^/NKG2A^-^) to immature CD56^+^NK cells (CD34^-^/CD117^+^/CD56^+/-^) precursor intermediate and finally a functional CD56^bright^ NK cell (CD34^-^/CD117^+/-^/CD94^+^). They attain full functional maturity by expressing the NKG2A or KIRs (CD34^-^/CD117^-^/CD94^+^/CD16^+^/KIR^+^) (45). Another marker of importance in the identification of mature NK cells is CD57. Cells expressing CD57 have high cytotoxic potential with low proliferation capacity. NK cells are the first immune cells to reconstitute post-transplant and are the primary cells to keep leukemia in check immediately post-HSCT. The normal maturation process is essential in conferring NK cells the ability to recognize and eliminate cancer cells. Mundy-Bosse et al. (46) demonstrated that the immature NK phenotype is upregulated in murine AML models, whereas the intermediate phenotype is almost absent. Chretien et al. (47) in their study divided the AML patients into three groups based on their NK maturation profile: hypo-maturation group with CD56^bright/dim^ KIRs^-^ CD57^-^; intermediate group with CD56^dim^ KIRs^-/+^ CD57^-/+^ and hyper-maturation group with CD56^dim^ KIRs^+^ CD57^+^ profile. Their study reported that the patients with the hypo-maturation profile had inferior outcomes, such as three-year OS and RFS. This study highlighted that the maturation status of NK cells plays a vital role in predicting long-term outcomes. Post-transplant infections such as cytomegalovirus (CMV) have also been implicated in influencing the reconstitution of mature NK cells post HSCT resulting in lower rate of relapse. Cichocki et al. reported that mature NK cells (CD56^dim^CD57^+^NKG2C^+^) predominantly expand in post HSCT recipients after CMV reactivation leading to better DFS (48). Armin et al. (49) showed that recipients with CMV reactivation had a higher frequency of KIR^+^NKG2A^-^ NK cells than in CMV seropositive recipients without reactivation and seronegative recipients. Hassan et al. (50) observed a rapid and sustained increase in NK cell numbers in patients with CMV reactivation, with an increase in the proportion of NKG2C expressing NK cells. Mundy-Bosse et al. (46) further showed that AML patients with defective NK cell maturation carrying lower levels of the transcription factors required for terminal NK differentiation (TBET and EOMES) was associated with elevated levels of miR-29b. These are important in controlling final NK cell differentiation. Moreover, lower T-bet expression leads to lower perforin in mature NK cells, which results in NK cells with reduced cytotoxic potential.

### 2.4 Expression of checkpoint inhibitors

Immune checkpoint molecules are essential in maintaining the immune response by protecting self-cells from lysis. Immune checkpoints like PD-1, TIM-3, and TIGIT regulate NK cell activity (51). PD-1 is expressed in mature NK cells when stimulated by infected cells or tumor cells. NK cells expressing PD-1 have reduced proliferative potential and lower cytotoxic and cytokine-producing capabilities. Blocking the interaction of PD-1 with its ligand PDL-1 by using specific antibodies activates NK cytolytic activity. PDL-1 expression in AML patients has been reported with lower immune recognition (52). Goltz et al. (53) showed that reduced PDL-1 expression in AML cells correlated with better outcomes, such as a lower risk of relapse and prolonged OS. Another checkpoint TIGIT is expressed on NK cells. The ligand for TIGIT is CD112, CD113 and CD155 same as that for DNAM-1. Sanchez-Correa et al. (54) showed low expression of DNAM-1 in AML patients, which could favor the binding of TIGIT to its ligands, sending inhibitory signals and leading to tumor evasion. Blocking TIGIT promotes NK cell-mediated cytotoxicity, as shown in cancer mouse models by Zhang et al. (55).

The above studies highlight the role of different NK cell defects in AML disease susceptibility and progression (**Figure 1**). Many of these studies formed the basis on which various targets for cell-based therapies were devised for AML.

### 2.5 Unfavorable tumor microenvironment and epigenetic modifications enabling tumor evasion

The tumor microenvironment (TME) is diverse, made up of stromal cells, immune cells, extracellular matrix and secreted factors (56). Interaction of AML blasts with the TME can be responsible for AML disease development, relapse, progression and resistance to therapy. Mesenchymal stem cells (MSCs) modulate the development and differentiation of hematopoietic stem cells, in the case of AML, the bone marrow MSCs can contribute to tumor progression by providing anti-apoptotic signals to AML blasts (57, 58). Regulatory T cell (Tregs) subsets are responsible for maintaining the peripheral homeostasis and central tolerance by suppressing T- helper (T_h_) cell proliferation. A few studies have reported increased numbers of Tregs population in AML patients (59, 60), and is associated with unfavorable treatment outcomes (61). Depletion of these tumor associated Tregs results in better cytotoxic T (T_c_) cell therapy (62). At the time of diagnosis NK cells in the AML patient are defective due to factors such as downregulation of activating receptors, upregulation of inhibitory receptors and lower cytotoxic and higher immature NK subsets (40, 47, 63). Paczulla et al. (64) showed that downregulation of the ligand for the NKG2D receptor (NKG2DL) *via* the PARP1 enables tumor evasion in AML blasts (64). Moreover, susceptibility of AML cells to NK cells is affected by the direct interactions between AML cells and mesenchymal stomal cells in the tumor microenvironment (65). Most common reasons for inhibition of NK activity in the TME are hypoxia, low glucose concentrations, cytokines, tumor cell derives factors such as IL6, IL10, TGF-Beta, prostaglandin E_2_ (PGE_2_). Myeloid derived suppressor cells (MDSCs) have an immunosuppressive role and expansion of MDSCs in AML patients leads to an immunosuppressive TME with a possible role in tumor progression (66–68). The expansion of MDSCs can take place through different pathways such as MUC1 oncogene expression through c-myc expression. Another group of cells in the TME is the Tumor associated macrophages (TAMs) which promote tumor progression. Al-Mataray et al. (2016) showed that AML promotes infiltration of TAMs (69). Further, AML blasts also secrete various immunosuppressive cytokine and chemokines such as IL-10, TGF B, IL-35 into the tumor microenvironment giving it an immune-inhibiting property (70–72).

Epigenetic modifications such as DNA methylation, histone modifications, Chromatin remodeling, control normal gene expression. Such epigenetic changes can also influence NK cell development, differentiation and functionality. DNA methylation involves transfer of a methyl group to 5’ position of cytosine molecule in CpG sites leading to normal gene expression. DNA methyltransferases (DNMTs) control these DNA methylation patterns. Nucleoside analogues of cytosine are integrated into the DNA and help in covalent bond formation with the DNMT, leading to DNA degradation and inhibition of DNA methylation (73, 74). Hypermethylation of the CpG islands can result in gene silencing. In case of tumor suppressor genes, hypermethylation promotes tumorigenesis (75, 76). Hypomethylating agents (HMAs) such as DNMT inhibitors and 5-aza-2’deoxycytidine (decitabine) have been used for at least a decade to treat high risk AML patients (77). Post translational modifications (PTMs) at the histone N-terminal tails, such as acetylation, methylation, phosphorylation, deamination etc. weakens the packed chromatin structure favoring the binding of transcription factors and normal genetic regulation. However, enzymes such as histone deacetylases (HDACs), demethylases, dephosphorylase reverse this function by removing the acetyl group from the histone tails leading to more compact packing of the DNA around the histones resulting in poor or no binding of transcriptional factors and hence affecting the gene transcription (78, 79). Such modifications of these epigenetic processes favor malignancy and tumor progression.

## 3 Therapeutic options for AML

### 3.1 Conventional therapies

Treatment for AML varies based on the stage of the disease and the type of mutation involved. Stem cell transplantation remains the most suitable treatment for AML patients to reduce the risk of relapse and mortality. However, the risk of relapse depends on several other factors, such as the blast clearance status in bone marrow, the presence of FLT3 mutations and cytogenetics, and molecular mutations, among others (80, 81).

Conventional treatment strategies for AML include chemotherapy, targeted therapy, and radiation treatment, with hematopoietic stem cell transplantation (HSCT) as the final treatment option. The most common chemotherapy regimens include standard protocol of 7 days of cytarabine and three days of anthracycline (82), fludarabine–Ara-C–granulocyte colony-stimulating factor–idarubicin (FLAG-IDA) for relapsed childhood AML (83), or similar kind of induction, followed by consolidation chemotherapy and hematopoietic stem cell transplantation in patients who are at a high risk of relapse. Although, these methods have resulted in complete remission in 60-80% of elderly patients the downside to this approach is induction-related mortality due to poor tolerance to these drugs (82, 84, 85).

HiDAC consolidation doses on days 1, 3, and 5 for younger patients not undergoing HSCT is a standard norm (86–88). Consolidation along with an added targeted drug results in more prolonged relapse-free survival (RFS) (89). Most AML patients achieve complete remission (CR) with intensive chemotherapy alone. However, many of those patients would relapse. In such a situation, hematopoietic stem cell transplantation (HSCT) becomes the treatment of choice.

### 3.2 Therapies targeting the tumor microenvironment and epigenetic modifications

In AML, the leukemic cells interact with the bone marrow microenvironment and secrete a cytokine called KIT ligand, which favors tumor progression by interfering with normal hematopoeisis (90–92). There have been studies to investigate the role of CXCR4/CXCL12 inhibitors, TGF-Beta neutralizing antibodies and use of monoclonal antibodies to block IL-6 (93–96). Hypoxia is another feature of the TME which favors the growth and proliferation of AML cells. Hypoxia downregulates the NK cell ligands like NKp30, NKp44, NKp46, NKG2D, perforin and granzymes. The NKG2D can be restored by administration of IL-2 (97). Hypoxia-activated prodrugs such as Evofosfamide and PR-104 have been shown to inhibit hypoxia associated resistance to therapy in AML (98–101). Glucose is a pre-requisite for generating ATP and NADPH in order for NK cells to function correctly, however, the TME is glucose deprived which impairs NK cell glycolytic activity. Some metabolic regulators like GLUTS have also been shown to regulate glucose influx (102). Recombinant cytokines such as Il2, Il-12, Il-18, IL-15, IL-21, IFN-gamma, GM-CSF have been shown to promote, mediate and enhance NK cell expansion and cytolytic potential (103–106). Although this therapeutic option of targeting different metabolites in the TME is fascinating, in AML the microenvironment is so complex that targeting a single metabolite to get the desired outcomes is a challenge.

Epigenetics is regulated by molecules such as DNA and histone methyltransferases (HMAs) and histone deacetylases (HDACs) (107). DNMT inhibitors dectabine and 5-aza-2’deoxycytidine (DAC) are known HMAs that have been approved for clinical treatment of AML and MDS. DAC is phosphorylated to decitabine triphosphate which then incorporates into the DNA and inhibits DNA synthesis causing a cytotoxic effect, whereas, 5-AZA gets integrated into the RNA structure and interferes with protein formation (108–110). There have been a few clinical trials investigating HMAs for treatment of AML. Issa et al. (111) have reported good efficacy and tolerance with lower doses of these drugs. In the first trials doses of 1500 to 2500 mg/m^2^ were used to stop DNA synthesis and cause cytotoxicity (112). Cashen et al. (113) in their Phase II clinical trial of decitabine treatment in elderly AML patients showed complete remission (CR) in 24% and overall response rate (ORR) in 25% with a median survival of 14 months. Lubbert et al. (114) treated their AML patients with decitabine + ATRA and observed ORR in 26%, with CR in 13.2% and PR in 12.8% with a median survival time of 5.5 months and 1 year OS of 28%. HDACs regulate cell apoptosis and proliferation by deacetylating lysine residues on proteins. HDAC inhibitors on the other hand inhibit this deacetylation process and help in controlling tumor progression. Some of the HDAC agents currently used are Valproic acid, Vorinostat and Entinostat among others. Vorinostat has a hydroxamic acid component which when binds to the zinc pocket of HDAC 1,2,3 and 6 results in reversible inhibition of HDACs and apoptosis of cancer cells (115).Valproic acid targets the AML1/ETO complex and induces apoptosis of AML cells (116). Entinostat helps in promoting loss of leukemia and longtime survival s proven in animal models (117, 118). Although by themselves HMA and HDAC inhibitors have shown moderate results combination therapies involving these molecules with other therapies may be promising.

### 3.3 Targeting the NKG2D-NKG2DL

Factors such as viral infections, oxidative damage, DNA damage increase the expression of metalloproteinases on tumor cells. Metalloproteinases have been suggested to be involved in the cleavage of NKG2D-L in AML (119–124). Raneros et al. (125) showed that azacitidine a DNA methyltransferase (DNMT) inhibitor reduces the release of soluble NKG2D-L in AML cells. Poly ADP ribose polymerase 1 (PARP1) inhibits the expression of NKG2D-L proteins in leukemic stem cells (LSCs) helping them evade surveillance (64). In case of DNA damage, the ATM/ATR pathway activates the PARP1 (126, 127), which in turn upregulates the expression of NKG2D-L on the surface of leukemic stem cells in AML (64). Nanbaksh et al. (128) reported that lower expression of c-Myc results in decreased expression of NKG2D-L in AML. Moreover, HDAC inhibitors and demethylating agents can also induce NKG2D-L in tumor cells making them susceptible to NK cells lysis (129–132). A few ongoing clinical trials are focused on targeting the NKG2D/NKG2DL axis in AML. CAR-NKG2D’s have been manufactured by introducing NKG2D into a CAR-T that will target the NKG2D-L in relapsed/refractory (r/r) AML patients (133–138). Baumeister SH et al. (134) confirmed the safety of such a CAR-T in a recent phase 1 clinical trial. These studies provide a new option for the treatment of AML through NK cells by targeting the NKG2D-NKG2D-L axis.

### 3.4 Hematopoietic stem cell transplantation

HSCT has been designated as the penultimate therapy for adult AML patients in CR1. It can reduce the risk of relapse by more than 60% compared to intensive chemotherapy alone by virtue of its potent graft-versus leukemia (GvL) effect (139). The advance of strategies, such as the use of post-transplant cyclophosphamide, has led to an increase in the number of suitable haplomatched donors for patients who do not find a fully matched HLA donor (140). However, even with changes in conditioning regimens (myeloablative to reduced intensity) and use of graft versus host disease (GvHD) prophylaxis such as cyclosporine, tacrolimus, mycophenolate mofetil, the success of HSCT is marred by post-transplant complications such as relapse, GvHD, and TRM. Hence, newer treatment options with fewer complications are sought. In recent years the role of KIR receptor-HLA ligand mismatch between the patient and donor favoring HSCT outcomes has gained much attention. **Table 1** highlights some of the published data on patient-donor KIR genotype, KIR ligand match/mismatch and KIR receptor ligand match on transplant outcomes. Most of these studies show the role of KIR B genotype in reduced risk of leukemia relapse and improved disease-free survival, lower GvHD, lower risk of CMV reactivation (9, 141–143). On the other hand, Bultitude et al. (144) showed higher non relapse mortality (NRM) in AML patients undergoing T cell depleted HLA-matched, unrelated donor HSCT where the donor carried the centromeric KIR B haplotype. Bao et al. (145) reported a higher rate of relapse in patients transplanted with graft from KIR A/A donors, whereas Mansouri et al. (146) reported similar results when the patient had a KIR A/A haplotype. Some reports suggest no role of KIR mismatch on transplant outcomes (147–149), whereas higher TRM, reduced OS and DFS has been reported by Kroger et al. (150), DeSantis et al. (151), in the presence of KIR mismatch transplants. On the other hand, favorable outcomes such as lower aGvHD (152, 153), lower risk of relapse (154–157) and better OS and DFS (158) has been reported by other studies. These contradictory results across studies could be due to the difference in study designs such as heterogenous population, HLA match/mismatch, donor being related/unrelated to the patient and the conditioning regimen used. These studies suggested some insights into the role of KIR and NK cells as the basis for immunotherapies.

**Table 1:** KIR genotype and HSCT in AML patients.

Variable	Study Design	Patients	Results	Reference
Donor with KIR B/X genotype	HLA matched and mismatched T cell replete transplants	448 AML patients	Better 3-year OS, lower risk of relapse, higher incidence of cGvHD	Cooley et al. (9)
Donor KIR B haplotype and patient expressing C1 epitope for HLA-C	RIC, unrelated donor transplantation	Prospective cohort n=243 Retrospective cohort n=2419	Reduced risk of leukemia relapse and improved disease-free survival	Weisdorf et al. (141)
Donor KIR B haplotype (KIR2DL2 or KIR2DS2 gene present)	HLA-identical adult sibling HSCT	134 AML patients	Longer relapse free survival	Impola et al. (8)
Donor KIR Centromeric B haplotype	HLA mismatched	65 patients with different hematological malignancies	Higher overall survival and relapse free survival and lower GvHD	Gautam et al. (142)
Donor with KIR B/x haplotype and 2DS1 activating receptor	HLA matched sibling/unrelated donor transplantation	288 AML patients	Lower risks of CMV reactivation	Nakamura et al. (143)
Donor centromeric KIR B	T cell depleted HLA-matched, unrelated donor HSCT, with MAC regime	119 AML patients	Higher Non relapse mortality (NRM)	Bultitude et al. (144)
Donors with KIR A/A haplotype and 2DS4*001 allele	T cell depleted unrelated donor transplants	75 patients with various hematological malignancies	Higher risk of aGvHD	Bao et al. (145)
Patients with KIR AA genotype and C2/Cx, Bw4+ (or A-Bw4+) or HLA-A3−/A11− genotypes who received HSCT from KIR Bx donors	HLA-matched siblings transplants	100 AML patients	Increased risk of aGvHD	Mansouri et al. (146)
KIR mismatch	T cell depleted mismatched unrelated donor transplants	24 patients with various advanced myeloid malignancies	No difference in aGvHD, cGvHD, relapse	Weisdorf et al. (147)
KIR mismatch	Unrelated HSCT	186 patients with various hematological malignancies	No difference in aGvHD, cGvHD, relapse	Sivula et al. (148)
KIR ligand mismatch	T cell replete/depleted haploidentical unrelated donor transplant	1571 patients with various hematological malignancies	No difference in TRM, aGvHD, cGvHD, relapse and DFS	Farag et al. (149)
KIR ligand mismatch, Donors with KIR AA haplotype	T cell depleted unrelated donor transplants	142 patients with various hematological malignancies	Higher TRM, reduced OS and DFS. Transplants with donors carrying KIR AA haplotype resulted in reduced risk of relapse	Kroger et al. (150)
KIR ligand mismatch	HLA Mismatched unrelated Donor transplant	104 patients with various hematological malignancies	Higher TRM and lower DFS and higher risk of graft rejection. In GvH direction led to higher prevalence of Grade III-IV aGvHD	De Santis et al. (151)
KIR ligand mismatch and KIR receptor ligand mismatch	Haploidentical HSCT with MAC regimen	79 AML patients	Decreased risk in aGvHD and relapse rate and better overall survival.	Zhang et al. (152)
KIR mismatch between patient and donor	HLA matched unrelated donor transplantation	90 high risk AML patients	Lower aGvHD	Davies et al. (153)
Patient missing KIR ligand	Unrelated HSCT	116 patients with various hematological malignancies	Decreased risk of relapse	Wu et al. (154)
Mismatched HLA C ligand	Haploidentical HSCT	74 patients with various hematological malignancies	Better disease free survival	Duan et al. (155)
KIR3DL-ligand mismatch	Cord blood transplants	2840 patients with AML, ALL and CML	CMV reactivation up to 100 days post CBT lead to lower risk of relapse	Yokoyama et al. (156)
Higher inhibitory KIR receptor-ligand match between patient and donor	HLA matched unrelated donor transplantation	2359 AML patients	Lower risk of relapse	Krieger et al. (157)
KIR ligand mismatch	T cell depleted haploidentical unrelated donor transplant	130 patients with various hematological malignancies	Better OS and DFS	Giebel et al. (158)
Donor Centromeric AA genotype	T-replete Haploidentical HSCT with PtCy	81 adult patients with different hematological malignancies	Lower incidence of relapse	Dubreuil et al. (159)
KIR ligand mismatch	T cell replete post Cy haploidentical HSCT	144 patients with various hematological malignancies	Lower risk of relapse, better disease free survival	Wanquet et al. (160)
KIR ligand mismatching	T cell replete haploidentical post Cy	444 patients with Acute lymphoid/myeloid leukemia	Worse overall survival	Shimoni et al. (161)
Donors with full-length KIR2DS4	Matched or 1 mismatch unrelated HSCT	75 patients with different hematological malignancies	Higher incidence of aGvHD	An et al. (162)
Donor KIR2DS1and patient with HLA C2	HLA mismatched sibling/unrelated donor transplants with MAC regimen	314 patients with mixed hematological malignancies	Improved overall survival and decreased risk of transplant related mortality	Tordai et al. (163)
Donor activating killer cell immunoglobulin-like receptors genes (KIR2DS1, KIR2DS3 or KIR3DS1)	Haploidentical HSCT	300 patients with mixed hematological malignancies	Increased EBV reactivation	Wang et al. (164)
Donor KIR2DS1	HLA haplo/mismatched T cell replete HSCT	91	Improved Overall survival in patients who were in complete remission at time of transplant	Ido et al. (165)
Patients with HLA C homozygosity (C1C1/C2C2)	HLA matched related/unrelated transplants	406 AML and MDS patients	Lower risk of relapse if early CMV reactivation occurred. Increased NRM and grade III-IV aGvHD	Nikoloudis et al. (166)
Patients homozygous for HLA B and HLA C epitopes	T cell replete HSCT, HLA matched/mismatched unrelated donors	1770 patients with various hematological malignancies	Lower relapse in HLA mismatched cohort	Hsu et al. (167)
Patients homozygous for HLA C epitopes (C1C1/C2C2)	HLA matched sibling donor transplant	52 patients with various hematological malignancies	Lower incidence of cGvHD. Higher OS and DFS	Wang et al. (168)

### 3.5 CAR-T therapies

Recently the focus has shifted to more and more adoptive cell therapies for treating leukemias. Chimeric Antigen receptor (CAR)-T cell therapy was the first cell therapy to receive FDA approval after showing success in patients with aggressive B cell malignancies. The idea that T cells could be used to target specific antigens was brought forward by Eshar et al. in 1989 (169). The use of CAR-T by engineering the patient’s self-cells to target CD19 which is an important target in ALL has been evaluated in various studies (170–172). CAR T cells secrete anti-tumor cytokines, perforin, and granzymes into the tumor microenvironment (TME) (173, 174). Generation of CAR-T involves high costs and takes a long time from generation to implementation as the patient’s self-cells are engineered. Various studies have evaluated the role of CAR-Ts targeted towards different AML antigens.

### 3.6 Monoclonal antibody targeted therapies

The cell surface expression of proteins differs on cancer cells compared to healthy cells. Cancer cells may over-express or under-express a surface protein and cause aberrant expression of proteins (175). CD33, CD45, CD123, and CD244, are some of the surface proteins ubiquitously expressed on AML blast cells (176). These molecules are promising targets for immunotherapy, and currently, there are many ongoing trials focused on developing monoclonal antibodies (mAbs) against these molecules to achieve better outcomes in AML patients. mAbs can be either naked antibodies that function through NK mediated ADCC, such as lintuzumab (anti-CD33) or mAbs conjugated to toxins (e.g., gemtuzumab ozogamicin – anti-CD33), or conjugated to radioactive particles (176).

### 3.7 NK cell-directed therapies for AML

Natural Killer (NK) cell immunotherapy is a novel immunotherapeutic treatment option. In AML patients, the NK cell function is suppressed, thus enabling the cancer cells’ to evade immune surveillance. NK cell immunotherapy prevents the suppression of NK cells so that they can carry out target lysis. Many clinical trials for various NK cell-based immunotherapies like adoptive NK cell transfer, chimeric antigen receptor (CAR) NK cells, cytokine-induced memory-like (CIML) NK cells, etc., are currently being carried out (2, 4).

**Figure 2:**
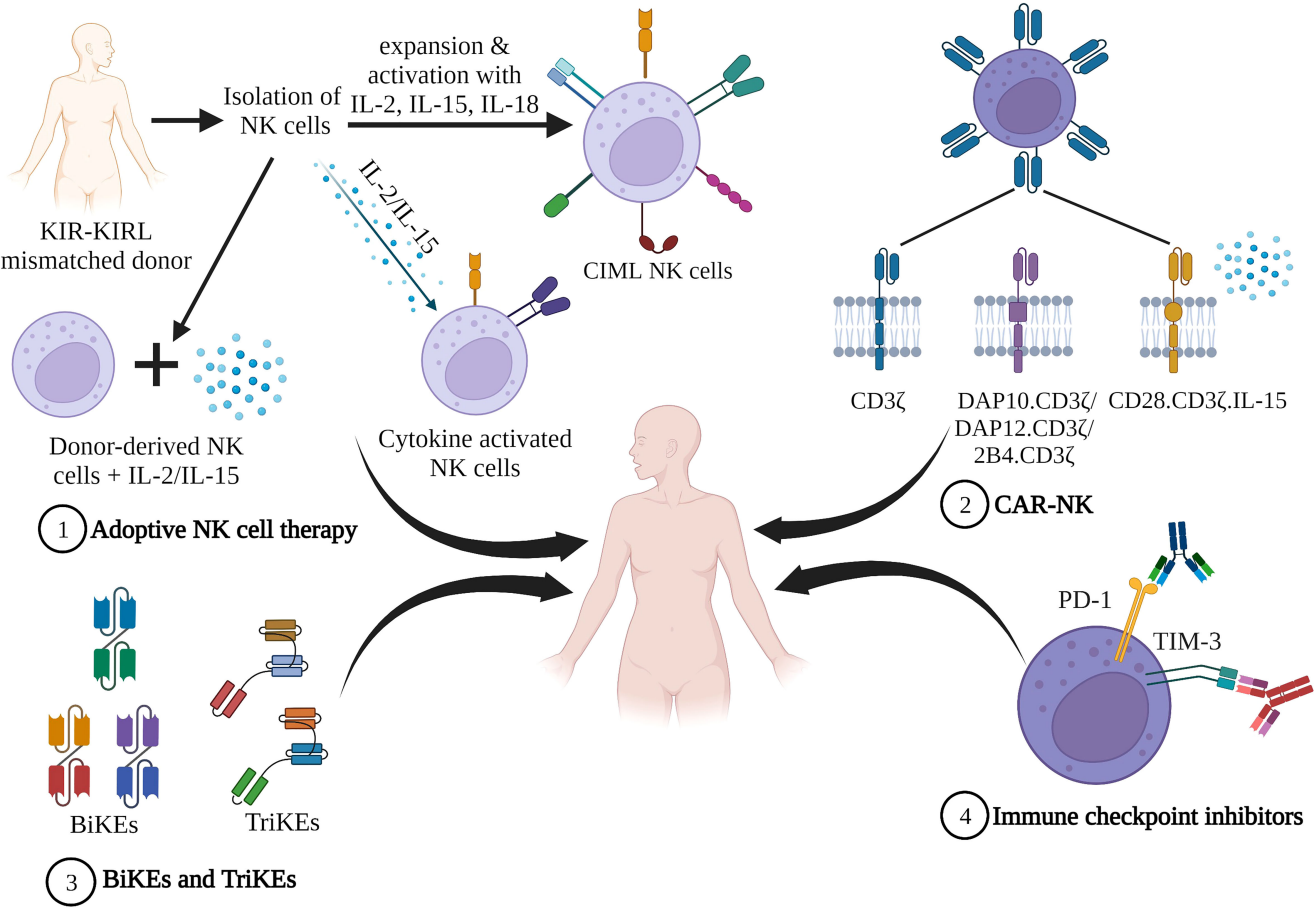
NK cell based immunotherapies. 1) Adoptive NK cell therapy. Infusion of cytokine stimulated ex vivo expanded donor derived NK cells 2) Infusion of CAR-NK construct that comprises an extracellular receptor recognizing a specific antigen, a hinge region, and a transmembrane domain and intracellular co-stimulatory domains. 3) NK cell engagers consisting of bi-specific killer engagers (BiKEs) and tri-specific killer engagers (TriKEs), comprised of a single variable antibody portion (V_H_ and V_L_) that links to either one (BiKE) or two (TriKE) variable portions on other antibodies. 4) Immune checkpoint proteins when bound to their ligands suppress NK cell activity facilitating tumor evasion. Blocking these checkpoint proteins with inhibitors prevents NK suppression (Figure created in BioRender).

NK cells lyse their target firstly by forming an immunological synapse with them, followed by the release of cytolytic granules and cytokines. The second mode of lysis is antibody-mediated cell cytotoxicity (ADCC), wherein NK cells recognize antigen-coated target cells by their FCgammaIIIA (CD16) receptor and trigger ADCC and cytokine production (177, 178). In 2012, Romee et al. (179) demonstrated memory like function of NK cells by showing that if NK cells are preactivated with IL12, IL15, and IL18 followed by 1-3 weeks’ rest, they were able to generate enhanced IFN-gamma production when exposed to cytokines or K562 leukemia cell line. These hypotheses led to the investigation of different cell sources that could be used to generate NK cells in adoptive cellular therapies. Some of the cell sources are cord blood, peripheral blood mononuclear cells (PBMC), cell lines such as NK-92, hematopoietic stem and progenitor cells, and induced pluripotent stem cells (179–188). There are advantages and disadvantages associated with every NK cell source used. Generation of NK cells from donor PBMCs is the safest and most favorable option as they carry various NK cell activating markers such as CD16, NKG2D, NKp44, and NKp46 which strongly recognize and kill non-self or tumor cells; however, the percentage of NK cells in peripheral blood is too low, and expansion of NK cells derived from PBMCs is costly and time-consuming (189–191). NK cells expanded from NK-92 cell lines, which are an immortalized NK lymphoma cell line, raises safety concerns and irradiation of products derived from these cell lines requires irradiation before infusion. NK-92 cell lines also lack various activating KIRs and Cd16 (FCRIII), which may limit their killing potential even through ADCC. The advantages of using NK-92 cell line-derived NK cells are the speed at which they multiply without the need for feeder cells and their ability to secrete higher amounts of perforin granzymes and cytolytic cytokines (192–195). Induced pluripotent stem cells are a good source for NK cells as they have a higher expansion rate; however, these, too, express lower CD16 levels, which could reduce their cytotoxic capacity, although this could be rectified by genetic engineering (196). Not only various sources of NK cells but also different NK cell-based immunotherapies such as adoptive transfer, CAR-NKs, bi-specific and tri-specific killer engagers (BiKEs and TriKEs) are recently being investigated in clinical trials to understand their role as a curative option for AML (**Figure 2**). **Table 2** lists the various completed clinical trials of NK cells in AML.

**Table 2:** NK-mediated clinical trials in AML.

Sr No.	Trial Number	Intervention	Number of Patients
1	NCT02395822^a^	A phase II trial of CD3/CD19 depleted, IL-15 activated, donor natural killer (NK) cells	17
2	NCT01385423^b^	A single-center, dose-escalation study designed to determine the maximum tolerated, minimum efficacious dose (MTD/MED) of IL-15 (Intravenous Recombinant Human IL-15) and incidence of donor natural killer (NK) cell expansion by day +14 when given after haploidentical donor NK cells in patients with relapsed or refractory acute myelogenous leukemia (AML).	26
3	NCT00187096^c^	Donor-recipient inhibitory KIR-HLA mismatched NK infusion post-multi-agent chemotherapeutic conditioning regimen (Cyclophosphamide, Fludarabine, Clofarabine, Etoposide, Interleukin-2) before the infusion	49
4	NCT00274846^d^ & NCT01106950^e^	Donor PBSC-derived natural killer (NK) cells (at a dose of 1.5-8 x 10^7/kg.) and IL-2 infusion post lymphodepletion with cyclophosphamide, fludarabine	21
5	NCT01947322^f^	Interventional Phase I/II AllogenicIL2 ex-vivo activated NK cells in 10 AML patients	10
6	NCT02316964^g^	A pilot trial involving decitabine, donor natural killer cells, and aldesleukin in treating patients with R/R acute myeloid leukemia	8
7	NCT01787474^h^	This phase I/II trial studies the side effects and best dose of donor natural killer cells in R/R AML	30
8	NCT00703820^i^	A Phase II Study Of Natural Killer Cell Transplantation In Patients With Newly Diagnosed Acute Myeloid Leukemia	324
9	NCT00303667^j^	Reduced Intensity Haploidentical Hematopoietic Stem Cell Transplantation (HSCT) Supplemented With Donor Natural Killer (NK) Cell Infusions in patients with high-risk myeloid malignancies	50
10	NCT03081780^k^	Open Label Dose Escalation Trial of an Adaptive Natural Killer (NK) Cell Infusion (FATE-NK100) With Subcutaneous IL-2	6
11	NCT00402558^l^	Alloreactive NK Cells With Busulfan, Fludarabine, and Thymoglobulin	15
12	NCT02763475^m^	Phase 2 Natural Killer (NK) cells as consolidation therapy	7
13	NCT02395822^n^	A phase II trial of CD3/CD19 depleted, IL-15 activated, donor natural killer (NK) cells in adults and subcutaneous IL-15	17
14	NCT03050216°	phase II trial of CD3/CD19 depleted, ALT-803 activated, haploidentical donor NK cells and subcutaneous ALT-803 given after lymphodepleting chemotherapy (CY/FLU)	8
15	NCT00900809^p^	Phase I trial of NK-92 as adoptive immunotherapy	7
16	NCT01795378^q^	Phase I/II trial of HLA-Haploidentical Hematopoietic Cell Transplantation and Subsequent Donor Natural Killer Cell Infusion	56
17	NCT00394381^r^	Phase I/II trial of Autologous Cytokine-induced Killer Cell Adoptive Immunotherapy	17
18	NCT01370213^s^	Phase II trial involving reduced intensity conditioning using Fludara, Cytoxan, and irradiation, followed by infusion of donor NK (natural killer) cells, interleukin-2 (IL-2) to promote NK expansion, ATG for additional immunosuppression to promote engraftment, and infusion of a TCR α/β-depleted same donor graft	25
19	NCT01904136^t^	Phase I/II trial to study the side effects and best dose of natural killer cells before and after donor stem cell transplant	90
20	NCT01823198^u^	Phase I/II trial studying the side effects and best dose of donor natural killer cells	63
21	NCT00460694^v^	Phase I/II trial infusion of allogeneic CIK cells	24

a https://clinicaltrials.gov/ct2/show/NCT02395822

b https://clinicaltrials.gov/ct2/show/NCT01385423

c https://clinicaltrials.gov/ct2/show/NCT00187096

d https://clinicaltrials.gov/ct2/show/NCT00274846

e https://clinicaltrials.gov/ct2/show/NCT01106950

f https://clinicaltrials.gov/ct2/show/NCT01947322

g https://clinicaltrials.gov/ct2/show/NCT02316964

h https://clinicaltrials.gov/ct2/show/NCT01787474

i https://clinicaltrials.gov/ct2/show/NCT00703820

j https://clinicaltrials.gov/ct2/show/NCT00303667

k https://clinicaltrials.gov/ct2/show/NCT03081780

l https://clinicaltrials.gov/ct2/show/NCT00402558

m https://clinicaltrials.gov/ct2/show/NCT02763475

n https://clinicaltrials.gov/ct2/show/NCT02395822

°https://clinicaltrials.gov/ct2/show/NCT03050216

p https://clinicaltrials.gov/ct2/show/NCT00900809

q https://clinicaltrials.gov/ct2/show/NCT01795378

r https://clinicaltrials.gov/ct2/show/NCT00394381

s https://clinicaltrials.gov/ct2/show/NCT01370213

t https://clinicaltrials.gov/ct2/show/NCT01904136

u https://clinicaltrials.gov/ct2/show/NCT01823198

v https://clinicaltrials.gov/ct2/show/NCT00460694

#### 3.7.1 Adoptive NK cell therapy

Adoptive NK cell therapy is the transfer of NK cells to a patient. The NK cells are isolated from a healthy donor (allogenic) or patient (autologous) using PB, bone marrow, or umbilical cord as the source. These NK cells are then either expanded or activated ex vivo using cytokines such as IL-2, IL-12, IL-15, IL-18, and IL-21 (197, 198). NK cells isolated from haploidentical donors show alloreactivity against leukemic cells due to KIR-KIR Ligand (KIR-KIRL) mismatch leading to the GvL effect (197). Currently, many ongoing clinical trials are investigating the efficacy of NK cell therapy in combination with various cytokines. Rubnitz et al. demonstrated the feasibility of donor-recipient inhibitory KIR-HLA mismatched NK cell transfer and the administration of IL-2 after an immunosuppressive regimen in 10 AML patients. Transient engraftment was observed in all patients, all patients were in remission at 2 years and the 2 year event free survival (EFS) was 100% (199).

Inpyo Choi et al. carried out a clinical trial to determine optimal dose of donor NK cell infusion (DNKI) to be administered in patients suffering from various hematological malignancies post HSCT (NCT00823524) (200). On receiving high doses of DNKI, no acute toxicity was observed in the patients, and a significant reduction was observed in post-transplant leukemia progression. Dolstra et al. (182) showed that an increased dose of NK cells generated from umbilical cord blood hematopoietic stem and progenitor cells (HSPC) was well tolerated in AML patients in CR and did not result in toxicity or GVHD.

Many studies involve the use of IL-2 along with NK cell infusion as IL-2 aids in stimulation of the infused NK cells (197). IL-2 is also responsible for the increased expression of the activating receptor NKG2D (190). Miller et al. (201) conducted a study to check the safety and efficacy of haploidentical donor NK cell infusion in AML patients who received either low-intensity or high-intensity immunosuppressive regimens. These patients were given a subcutaneous injection of IL-2 after NK cell infusions. Successful *in vivo* expansion of donor NK cells was observed in these patients with 60% patients achieving CR. At low doses, IL-2 can stimulate NK cells as well as the T_reg_ of the host, which can affect the NK cell function, whereas high doses can cause toxicity. As an alternative treatment, the use of IL-15 has been considered (202). In a first-in-human trial, recombinant human IL-15 (rhIL-15) was administered intravenously (NCT01385423) or subcutaneously (NCT02395822) to R/R AML patients after haploidentical NK cell transfer. Compared to previous studies using IL-2, it was observed that *in vivo* NK cell expansion was better when rhIL-15 was used. However, cytokine release syndrome (CRS) was seen in patients who received rhIL-15 subcutaneously (203). In the phase I/II trial, high doses of membrane-bound IL-21 (mb-IL21) ex vivo expanded donor-derived NK cells (NCT01904136) resulted in lower 2-year relapse rate with better disease-free survival (DFS) in patients suffering from various myeloid malignancies (204).

Recently, the use of memory NK cells for the treatment of leukemia has been gaining importance. It has been observed that NK cells activated in response to interleukins like IL-12, IL-15, and IL-18 exhibit memory cell-like characteristics. These cytokine-induced memory-like NK cells (CIML NK), also called memory-like NK cells (ML NK cells), display better effector function (103, 205). Many clinical trials are being carried out to explore CIML NK as an effective immunotherapy against AML (206). Shapiro et al. (207) reported that CIML NK cells expanded *in vivo* within 30 days of infusion in patients who have relapsed after haploidentical HSCT (NCT040247761). The leukocyte and granulocyte chimerism were high post-CIML NK infusion. Bednarski et al. (208) showed that the adoptive transfer of ML NK cells has no significant toxicity. These ML NK cells display potent cytotoxicity against leukemic cells and demonstrate long-term *in vivo* persistence.

#### 3.7.2 Monoclonal antibody therapy

Recently, a few clinical trials have been designed to explore the efficiency of killing by NK cells through ADCC using monoclonal antibodies (**Table 3**).

**Table 3:** Various ongoing clinical trials using monoclonal antibodies against different surface antigens.

Trial No.	Antibody	No. of participants	Intervention/Treatment	Phase	Status
NCT03441048a	Lintuzumab-Ac225 (anti-CD33)	26	CLAG-M chemotherapy with Lintuzumab-Ac225	1	Active, not recruiting
NCT05077423s	CD33*CD3, a bispecific antibody (BsAb)	36	Subcutaneous administration of CD33*CD3 BsAb up to 12 cycles	1	Recruiting
NCT04714372c	Daratumumab (anti-CD38)	50	Daratumumab given subcutaneously along with FT538, an off the shelf allogeneic NK cell immunotherapy	1	Recruiting
NCT05266274d	CD47 mAb	69	CD47 monoclonal antibody combined with azacitidine	–	Recruiting
NCT03248479e	Magrolimab (anti-CD47)	258	Magrolimab + Azacitidine	1	Active, not recruiting
NCT04227847f	SEA-CD70 (anti-CD70)	140	SEA-CD70 with and without azacitidine	1	Recruiting
NCT02730312g	XmAb14045 (Anti-CD123)	120	Intravenous administration of XmAb14045 weekly for up to 8 weeks, with or without step-up dosing	1	Completed

a https://clinicaltrials.gov/ct2/show/NCT03441048

b https://clinicaltrials.gov/ct2/show/NCT05077423

c https://clinicaltrials.gov/ct2/show/NCT04714372

d https://clinicaltrials.gov/ct2/show/NCT05266274

e https://clinicaltrials.gov/ct2/show/NCT03248479

f https://clinicaltrials.gov/ct2/show/NCT04227847

g https://clinicaltrials.gov/ct2/show/NCT02730312

#### 3.7.3 CARs

There are four generations of CARs that are currently being investigated: first generation-CARs comprising of the basic structure and one signaling region, second generation-CARs with an extra co-stimulatory domain, third generation –CARs with multiple co-stimulatory domains, and fourth generation-CARs with multiple co-stimulatory domains and cytokine signals (209–214). A CAR construct comprises a synthetic extracellular receptor that will recognize the specific antigen, a hinge region, and a transmembrane domain and may carry one (first generation) (209) or multiple (2^nd^, 3^rd^ generation) (210–213) intracellular co-stimulatory domains. CARs are fusion proteins expressed on immune cells so that these cells recognize specific targets. Initially, the CARs used for CAR-T therapy comprising CD3 ζ and the T co-stimulatory molecule were used for generating CAR-NK cells and evaluating their efficacy against tumors (215). Although there have been promising results, CAR-T therapy has been associated with side effects such as cytokine release syndrome (216–218), whereby an increased production of IL6, IFN-gamma, GM-CSF, and TNF-alpha result in severe neurological conditions, myalgia, hypoxia, hypotension, and vascular leakage (219). On-target/Off-tumor is another complication associated with CAR-T therapy (217, 220–222). The manufacturing process for CAR-T cells takes about 2-3 weeks post-patient cell accrual. Due to these limitations, researchers have started evaluating other immune cells for adoptive cell therapies. NK cells bind only to their cognate human leukocyte antigen (HLA) ligands on leukemic cells hence taking care of the toxicities that could occur due to nonspecific recognition of healthy cells. This power of distinguishing between self and non-self cells is conferred by the various activating and inhibitory receptors present on the NK cell surface (223, 224). NK cells also do not cause GvHD. These properties of NK cells have made them the cells of choice for adoptive therapies as they overcome most of the problems associated with CAR-T cell therapy.

Although, transfusion of unmodified NK cells has shown some efficacy in AML patients, such NK cells have a short lifespan leading to lower success rates, hence, the focus has shifted to CAR-NK cells, and their efficacy is being tested in various clinical trials. The success received from earlier studies then led to the generation of 2^nd^ and 3^rd^ generation CAR-NKs which utilizes the unique properties of NK cell stimulatory receptors such as DAP12, DAP10, and 2B4 (225–227). DAP10 and DAP12 are intracellular signaling domains and activated molecules such as NKp44, activating KIRs, and NKG2C (226, 228). DAP12 has ITAMs like KIRs, which, once phosphorylated, initiate the release of proinflammatory cytokines such as TNF-alpha and IFN-gamma (226). DAP10, on the other hand, does not carry an ITAM motif but induces potent NK cytotoxicity by signaling through the NKG2D-DAP10 axis (225). Both DAP10, along with the CD3 ζ CAR NK constructs and 2B4 with anti-CD5 CAR NK constructs, have shown promising results in B-ALL and T-ALL, respectively (228, 229). This was then expanded to include multi-specific target strategies (4^th^ generation), such as NKG2D CAR-NK cells which target tumors expressing ligands for NKG2D. The first success of CAR-NK cells in AML was reported by Tang et al. in 2018 (230). In their first-in-man clinical trial (NCT02944162), they tested the safety and efficacy of third generation CD33 directed CAR NK construct with CD28 and 4-1BB co-stimulatory domains using NK cells derived from NK-92 cell lines in 3 relapsed/refractory AML patients. The dosage used was 5x 10^9^ cells. None of the participants had adverse effects; however, there was no durable remission. The main limitation of this study was the short lifespan of the irradiated NK92 cell lines, with the number of CAR-NKs depleting to below measurable levels in 1-week post-infusion. Since there were no unfavorable effects, it was established that functional CAR-NK92 cells could be produced at a much lower cost than the CAR-T cells; however, low numbers post-infusion would still remain a problem. In preclinical studies, Christodoulou et al., 2021 (231) generated CARs with 2B4.ζ or 4-1BB.ζ signaling domains. These CAR NKs were tested for their cytotoxic activity *in vitro* and xenograft mouse models. It was observed that these CAR NKs show higher anti-AML cytotoxic activity *in vitro*; this activity could be enhanced by transient expression of secretory IL-15; however, only the 2B4.ζ Chimeric Antigen Receptor (CAR)-NK cells exhibited transient anti-AML activity in mice models. Further, in the *in vivo* 2B4.ζ/sIL-15 CAR-NK cells experiments, one set showed potent anti-AML activity, whereas the other set showed lethal toxicity. Morgan et al., 2021 (232) used alpha-retroviral vectors to modify NK92 cells and designed a third-generation CAR NK to target CD123, which is strongly expressed on the surface of AML cells. The vector also carried a transgene cassette to allow constitutive expression of human IL15, which ensures enhanced NK persistence *in vivo*. Salman et al. (233) developed a third-generation CAR construct (CD28-4-1BB- CD3 ζ) with CD4 as a target and tested them against AML in a cell line-derived xenograft mice model. Their study showed specific elimination of primary CD4 positive AML blasts and suppression of disease progression. Quite recently, Mezger’s group from Germany transduced NK-92 cells with CD276 CAR. Simultaneously using CRISPR technology, they introduced gene knock-out of the NK inhibitory checkpoints: CBLB, NKG2A, TIGIT) to enhance NK cytotoxicity. They tested the cytotoxic capacity of these cells against various leukemic cell lines. When they compared the cytotoxic potential between the two groups, there was a significantly higher cytotoxic potential CRISPR-Cas9 knock-out compared to the parental NK-92 cells. It was also observed that the triple knock-out CD276-CAR-NK-92 cells, as well as single CBLB or TIGIT knock-out NK-92 cells, showed significantly superior cytotoxicity against U-937 or U-937 CD19/tag AML cell lines. These results conclude that knock-out cells could be used as promising off-the-shelf therapeutic options for treating AML (234). Another target for NK CAR is NKG2D which interacts with various stress ligands expressed on abnormal cells. *In vivo* experiments with such NKG2D-directed NK, CARS have shown promising anti-leukemic response (235) and have now moved on to phase I clinical trials in R/R AML patients (NCT04623944). Such off-the-shelf therapeutics can reduce the cost and time required for NK cell therapies. Garrison et al. (236) used a data-driven bioinformatics approach to devise logic-gated CD33-OR-FLT3-NOT-EMCN CAR NK cells. The novel idea behind this approach is that such constructs will specifically target AML blasts and leukemia-initiating cells as they carry both CD33 and FLT3 markers; however, normal hematopoietic stem cells will be unharmed as they carry the endomucin (EMCN) marker. This hypothesis was proven successful in co-cultures. Over the past few years, researchers have constantly been working on developing safer and more effective CAR-NK therapy for AML by using different targets, different sources of NK cells, and different types of CAR constructs. **Table 4** shows the clinical trials currently in recruiting stage, where different CAR-NK cells are being tested against AML.

**Table 4:** Various ongoing studies investigating various engineered CAR-NKs in AML.

Trial Number	Intervention/Treatment	Number of participants	Details	Phase	Status
NCT05215015^a^	Biological: Anti-CD33/CLL1 CAR-NK cells	18	Administration of CAR-NK on day one and day3 of each cycle with the first dosage of 2.0×10^9 cells. Second administration dose in the first cycle 3.0×10^9 cells, and each dose in the second cycle 3.0×10^9 cells.	Early Phase I	Recruiting
NCT05574608^b^	Biological: CD123-CAR-NK cells	12	CD123-CAR-NK cells CD123-CAR-NK is an allogenic CD123-Targeted chimeric antigen receptor NK-cell (CAR-NK) therapy.	Early Phase 1	Recruiting
NCT02944162^c^	Biological: anti-CD33 CAR-NK cells Construct: CAR33-CD28-4-1BB- CD3 ζ	10	The allogeneic NK cells (NK-92 cell line for clinical use) are engineered to contain anti-CD33 attached to TCRzeta, CD28, and 4-1BB signaling domains. Enrolled patients will receive CAR-NK cells immunotherapy with a novel specific chimeric antigen receptor targeting CD33 antigen by infusion.	Phase 1Phase 2	Recruiting
NCT04623944^d^	Biological: NKX101 - CAR NK cell therapy Construct CAR.NKG2D-OX40- CD3 ζ (NKX101)	90	NKX101 - CAR NK cell therapy NKX101 is an investigational allogeneic CAR NK product targeting NKG2D ligands on cancer cells.	Phase 1	Recruiting
NCT05247957^e^	Biological: CAR-NK cells	9	NKG2DL-specific CAR-NK cells, two infusions on Day 0 and Day 7. After preconditioning with chemotherapy, NKG2DL-specific CAR-NK cells will be evaluated.	Phase 1	Recruiting
NCT05008575^f^	Biological: anti-CD33 CAR NK cells Drug: Fludarabine Drug: Cytoxan	27	anti-CD33 CAR NK cells 6×10^8, 12×10^8, 18×10^8/KG Treatment following lymphodepletion	Phase 1	Recruiting
NCT05601466^g^	Drug: QN-023aDrug: Cyclophosphamid Drug: FludarabineDrug: Cytarabine	18	Drug: QN-023a NK cell therapy	Phase 1	Recruiting
NCT05092451^h^	Drug: CyclophosphamideDrug: CAR.70/IL15-transduced CB-NK cellsDrug: Fludarabine phosphate	94	Cord blood source of stem cells. Construct CAR.CD70-IL15	Phase 1Phase 2	Recruiting
NCT02742727^i^	Biological: anti-CD7 CAR-pNK cells	10	CAR-pNK Cell immunotherapy Enrolled patients will receive CAR-pNK cell immunotherapy with a novel specific chimeric antigen receptor targeting CD7 antigen by infusion.	Phase 1Phase 2	Recruiting
NCT04023071^j^	hnCD16 CAR-NK against target CD20 (CD16a+ Rituximab)		NK cells from the iPSC source are used to generate these constructs against R/R AML and B-NHL.		Recruiting
NCT04614636^k^	CD38/SLAMF7 (CD16a+ Daratumumab/Elotuzumab)		NK cells from the iPSC source are used to generate these constructs against R/R AML, Multiple Myeloma.		Recruiting

a https://clinicaltrials.gov/ct2/show/NCT05215015

b https://clinicaltrials.gov/ct2/show/NCT05574608

c https://clinicaltrials.gov/ct2/show/NCT02944162

d https://clinicaltrials.gov/ct2/show/NCT04623944

e https://clinicaltrials.gov/ct2/show/NCT05247957

f https://clinicaltrials.gov/ct2/show/NCT05008575

g https://clinicaltrials.gov/ct2/show/NCT05601466

h https://clinicaltrials.gov/ct2/show/NCT05092451

i https://clinicaltrials.gov/ct2/show/NCT02742727

j https://clinicaltrials.gov/ct2/show/NCT04023071

k https://clinicaltrials.gov/ct2/show/NCT04614636

Even with all the advantages of using NK CAR therapies, this technology carries a few disadvantages. The first drawback is the low numbers and short lifespan of NK cells, approximately 1-4 weeks. However, incorporating cytokines such as IL2 and IL15 during the construction of CAR-NKs can improve the expansion and persistence of NK cells post-infusion (237). Newer ex-vivo expansion strategies have been used to generate more functional NK cells that could be used in CAR-NK therapy. These expansion methods involve culturing NK cells with cytokines such as IL15 and IL2, which aid in promoting NK expansion post-infusion and maintaining homeostasis. Large-scale expansion protocols use feeder cells such as Jurkat cell lines and the Epstein Barr virus-transformed lymphoblastic cell lines (EBV-LCL). However, it is yet to be seen which expansion methods can result in GMP-grade NK cells with high expansion potential and sustained numbers post-infusion, along with enhanced cytotoxic activity (238).

Another drawback is that most tumor cells have antigen loss or downregulation of these antigens, evading immune surveillance by CAR-NKs. Researchers have come up with various strategies to combat this issue. One strategy is to target two antigens so that the tumor cell will be detected. This can be done by generating a construct that can encode two different CARs, each with specificity for one antigen or a single CAR with two different recognition domains (239). Further, NK CARs are difficult to engineer due to their higher chances of apoptosis and low gene expression. Gene manipulation in NK cells is currently performed using electroporation which results in rapid transient expression, or by using viral vectors. However, these methods are still under development and need to be optimized for the widespread use of CAR-NK therapies in specific cancers (240, 241). Yet another limitation to CAR-NK-based therapies is that these can detect antigens only when they are expressed on the surface of the cells. Intracellular antigens are presented by MHC molecules and detected by TCRs. Taking advantage of this fact, Dr. Walchli and Dr. Inderberg edited the NK cell to display anti-cancer TCRs on their cell surface. They successfully created a functional NK-CAR expressing a TCR, which could kill cancer cells (242). Although these modified products hold a promising future as therapeutics, they are still in the validation phase, and their potential is yet to be proven.

#### 3.7.4 BiKEs and TriKEs

Although CAR-NKs have been showing promising results in preclinical and early clinical studies, these are expensive, time-consuming, and difficult to expand on a large scale. Hence, newer molecules known as NK cell engagers have recently gained interest as NK-based therapeutics. These molecules consist of bi-specific killer engagers (BiKEs) and tri-specific killer engagers (TriKEs), which are small molecules comprised of a single variable antibody portion (V_H_ and V_L_) that links to either one (BiKE) or two (TriKE) variable portions on other antibodies. In the case of BiKEs, one of the variable antibody domains recognizes CD16 and the other a targeted tumor antigen (242), whereas, TriKEs, in addition to these two domains, have an interleukin 15 (IL15) element that connects the two different antibody domains. This IL15 moiety enhances proliferation, persistence, cytokine production, and NK cytolytic potential, as proven in *in-vitro* assays with AML blast cells (243).

Additionally, these molecules may also have a tetra-specific design which can bind to multiple antigens on tumor cells or cross-link with cytokine units to favor NK expansion (243–247).

CD16A on NK cells is a receptor for Fc fragment of IgG antibodies. BiKEs engage CD16 activating receptors present on the NK cells by one of their variable fragments and a target antigen on the tumor cell by the other variable fragment. CD16A carries an ITAM which is phosphorylated upon binding of the CD16A to the antigen bound IgG. This phosphorylation results in inducing a kinase dependent signaling cascade leading to release of cytokines and cytolysis. Such molecules take care of the major difficulties faced with CAR molecules, such as there is no need to engineer these molecules and no requirement for gene transfer, which makes the production and expansion of these molecules much cheaper. Moreover, introduction of IL15 in TriKEs has been shown to improve *in-vivo* NK cell expansion and tumor control in mice models, taking care of the limitation of low NK cell numbers in the patient (243).

For AML, bi-specific reagents involve a construct of CD33, whereas tri-specific reagents involve a construct of CD33 and CD123 on AML (248–250). Wiernek et al. (251) created a BiKE against AML. This construct comprised a domain-specific for CD16 on NK cells and CD33 on AML cells. This was known as the 1633 BiKE. They, along with Gleason et al. (252), tested the potential of this construct *in vitro*. The results indicated that such a BiKE could overcome the inhibitory KIR signaling and stimulate NK cells to kill AML blasts, and stimulation of NK cells with such BiKEs *in vitro* restore the inhibited NK cell function in MDS patients. Valera et al. in 2016 (243) built the first TriKE by improvising the already existing BiKE 1633. They added IL15 to the BiKE and called it 161533, wherein the TriKE facilitates the formation of a synapse between the NK cell (CD16) and the tumor cell (CD33), binding to the CD16 on the NK cells to trigger ADCC and the IL15 will promote NK cell activation and expansion. Wiernek et al. (251) improvised on these TriKEs by co-administering ADAM17 inhibitors, which could help sustain CD16 expression on NK cells. Arvindham et al. (247) recently created a TriKE targeting CLEC12A on AML cells with anti-CD16 single-domain antibody and IL-15. Their results showed a potent NK cell-mediated response against primary patient-derived AML blasts. Another TriKE molecule developed against R/R AML is the GTB-3550 molecule currently in Phase 1/2 trial (NCT03214666). The interim data is promising, with a significant reduction in leukemic blasts in AML patients without adverse side effects such as cytokine release syndrome (253, 254).

There are issues faced with BiKEs and TriKEs, too, as these molecules are still in the early development phase. The use of IL15 in generating TriKEs may also pose a risk for stimulation of T cells resulting in cytokine release syndrome. Another issue similar to CAR-NKs is that TriKEs can kill healthy cells, too, as some of the tumor target antigens can be present on healthy cells too. Further, the effectiveness of such therapies is based on the binding of CD16 of the NK cell with the Fc portion of the antibody. However, mechanisms such as clipping of CD16 by matrix metalloproteins such as ADAM-17 can lead to diminished ADCC. Wernick et al. (251) provided a solution to this problem in their *in vitro* studies which showed that introducing ADAM-17 inhibitors with such therapies enhances cytolysis by BiKE against myeloid target cells. Another solution could be to target receptors other than CD16 on NK cells, such as NKG2D and 2B4, which have been proven to show similar activation as that compared to CD16 activation alone (255). It is too early to comment on the long-term survival, expansion, cytolytic activity, and safety of using these molecules for NK-mediated immunotherapies, and in-human trial data is warranted.

#### 3.7.5 Immune Checkpoint inhibitor blockade therapy

Newer strategies for NK immunotherapy target immune checkpoints, inhibition of which leads to tumor evasion. In relation to AML, the NK cell receptors such as KIRs and molecules such as TIM-3, CD200R, and others are currently being investigated for their potential in NK-mediated checkpoint inhibitor therapy. Two IgG4 monoclonal antibodies, IPH2101 and IPH2102 (lirilumab), that target KIR2DL1/2/3 NK inhibitory receptors are presently being investigated for single drug/combination therapy (256, 257). Romagne et al. (258), in their *in vivo* experiments, showed preclinical efficacy of blocking IPH2101 in AML cells. This efficacy was further proven in clinical trials by Vey et al. (257), where comparatively better clinical efficacy was evident in AML patients [NCT01256073, NCT01222286]. In a Phase II efficacy study, administration of Lirilumab did not show a significant difference when compared to placebo [NCT01687387] (259); however, when used in a combination therapy along with azacytidine, it was well tolerated in high-risk R/R AML patients (260).

TIM-3 is a co-inhibitory receptor that recognizes galectin-9 as a ligand. Binding TIM-3 to its ligand induces immune tolerance due to NK cell exhaustion, making it a negative regulator of NK cell immunity. Blocking TIM-3 has been shown to reverse NK cell dysfunction in various diseased conditions (261–265). Recently, an ongoing randomized phase I clinical trial investigating the efficacy of MBG453, an anti-TIM3 antibody alone or in combination with decitabine/spartalizumab/MBG453+decitabine+spartalizumab is underway [NCT03066648]. Another inhibitory receptor expressed on NK cells is CD200R which binds to its ligand CD200 expressed on various tumor tissues. Atfy et al. showed that overexpression of CD200 led to suppressed NK cell anti-tumor activity in AML patients resulting in an increased risk of relapse (266). Coles et al. (267) showed that blocking CD200 restored the NK activity to normal levels. A few other clinical trials investigating blockage of checkpoint inhibitors in AML, such as NCT03248479 [Magrolimab alone or in combination with azaticidine] and NCT03922477 [Magrolimab plus atezolizumab], are still in recruiting phase, and the results are yet to be seen.

## 4 Future directions

Over the years our understanding of NK biology and its role in designing anti-leukemic therapies has drastically improved. It is a known fact that leukemic patients most often carry NK cells that are dysfunctional/defective/low in numbers. Adoptive NK cell infusions have proven useful in restoring these functions albeit transiently. Recently, the focus has shifted from CAR-T and antibody therapies to NK cell-based immunotherapy due to the higher manufacturing cost and lower safety of T cell-directed therapies. CAR-NKs also exhibit fewer neurological toxicities and cytokine storm than CAR-T cell therapies.

Researchers are investigating cytokine-stimulated NK cell transfer, TriKEs, and BiKEs that boost NK functions and lead to longer proliferation of NK cells post infusion and hence longer periods of remission. Further, antibodies targeting immune checkpoints such as TIGIT, PD-1, and TIM-3 have theoretical potential, as blocking these checkpoints with specific antibodies helps in reversing the diminished NK cytolytic activity in AML patients. A few clinical trials are exploring the potential of combination therapies focusing on the infusion of the NK cell product and conventional treatments. NK cell therapies have proven their dominance over other cellular therapies due to their non MHC restricted recognition of tumor cells, potential to cure wider population, cost-effective manufacturing off the shelf that can be easily expanded *in vivo* and personalized for every patient. However, most of these studies are still in the preclinical and clinical stages, and as such, more extensive studies with reproducible results on methods of expansion, cryopreservation, infusion protocols, and safety are required to validate these therapies and make them a reality.

## Author contributions

The authors confirm contribution to the paper as follows: Concept: MS and SD’S. Draft manuscript preparation: SD’S and AP. Approving and Finalizing manuscript: SD’S and MS. All authors contributed to the article and approved the submitted version.

## Glossary

**Table:**

ADCC	antibody-mediated cell cytotoxicity
AML	Acute Myeloid Leukemia
BiKEs	bi-specific killer engagers
CAR	Chimeric antigen receptor
CIML	cytokine-induced memory-like
CMV	Cytomegalovirus
CR	complete remission
DFS	disease-free survival
DNKI	donor NK cell infusion
DNMT DNA	methyltransferase
EBV-LCL	Epstein Barr virus-transformed lymphoblastic cell lines
EMCN	endomucin
FLAG-IDA	fludarabine–Ara-C–granulocyte colony-stimulating factor–idarubicin
GvHD	graft versus host disease
GvL	graft-versus-leukemia
HDAC	histone deacetylases
HLA	Human leukocyte antigen
HMAs	Hypomethylating agents
HMT	histone methyltransferases
HSCT	Hematopoietic Stem Cell Transplantation
HSPC	hematopoietic stem and progenitor cells
ITAM	immune receptor tyrosine-based activating motifs
ITIM	immune receptor tyrosine-based inhibitory motifs
KIR	Killer immunoglobulin-like receptors
mAbs	monoclonal antibodies
mb-IL21	membrane-bound IL-21
MDSCs	Myeloid derived suppressor cells
ML NK	memory-like NK
MSC	Mesenchymal stem cell
NCR	natural cytotoxicity receptor
NK	Natural Killer
NKG2D-L	Ligand for the NKG2D receptor
OS	overall survival
PARP1	Poly ADP ribose polymerase 1
PBMC	Peripheral blood mononuclear cells
PGE_2_	Prostaglandin E_2_
PTMs	Post translational modifications
TAMs	Tumor associated macrophages
T_c_,	cytotoxic T cell
T_h_	T-helper
TME	Tumor microenvironment
Tregs	Regulatory T cell
